# Severe Euglycemic Ketoacidosis Secondary to Tirzepatide Use for Weight Loss

**DOI:** 10.7759/cureus.107028

**Published:** 2026-04-14

**Authors:** Alhassan Hassan

**Affiliations:** 1 Intensive Care Unit, Mid Cheshire Hospitals NHS Foundation Trust - Leighton Hospital, Crewe, GBR; 2 Emergency Department, Mersey and West Lancashire Teaching Hospitals NHS Trust - Whiston Hospital, Liverpool, GBR

**Keywords:** critical care medicine, euglycemic ketoacidosis, glp-1 receptor agonists, renal replacement therapy, starvation ketoacidosis, tirzepatide, weight loss complications

## Abstract

Numerous reports have documented the incidence of euglycemic ketoacidosis as a complication of sodium-glucose cotransporter-2 (SGLT-2) inhibitors. However, euglycemic ketoacidosis secondary to the use of glucose-dependent insulinotropic polypeptide (GIP) and glucagon-like peptide-1 (GLP-1) receptor agonists, such as tirzepatide, is becoming increasingly prevalent. While cases of euglycemic ketoacidosis in patients using tirzepatide managed with intravenous dextrose have been reported, this case describes a unique and severe presentation. We report a case of starvation-induced euglycemic ketoacidosis secondary to tirzepatide use resulting in transient neurological deficits, profound metabolic acidosis, and acute renal failure requiring intensive care admission and continuous renal replacement therapy. This report highlights the diagnostic challenges in patients presenting with altered levels of consciousness and an unclear clinical history, emphasizing the need for a high index of suspicion for ketoacidosis in patients on tirzepatide, even in the presence of normal blood glucose levels.

## Introduction

Sodium-glucose cotransporter-2 (SGLT-2) inhibitors have been associated with euglycemic ketoacidosis (EKA) as a known complication, but there are increasing reports of episodes of EKA with the use of glucagon-like peptide-1 (GLP-1) and glucose-dependent insulinotropic polypeptide (GIP) receptor agonists for weight loss [1,2]. Several agents in these classes have been approved by the US Food and Drug Administration (FDA) for clinical use. These include GLP-1 receptor agonists such as liraglutide and semaglutide, approved for the treatment of type 2 diabetes mellitus and chronic weight management, as well as tirzepatide, a dual GIP/GLP-1 receptor agonist approved for type 2 diabetes and, more recently, chronic weight management. These medications are being increasingly used for weight loss purposes with relatively easy access in the community.

Euglycemic ketoacidosis is diagnosed by the presence of ketonemia, high anion gap metabolic acidosis (HAGMA), and euglycemia (blood glucose level < 13.9 mmol/L) [3]. HAGMA is characterized by arterial pH < 7.3, bicarbonate (HCO3) less than 18 mmol/L, and a raised anion gap. Some precipitating factors for the development of EKA include infection, surgery, fasting, alcohol intake, acute vascular events, and trauma [4].

Tirzepatide can precipitate EKA by significantly delaying gastric emptying and suppressing appetite. This leads to a state of 'masked' starvation where low carbohydrate intake and reduced insulin levels promote lipolysis and ketogenesis, with serum glucose remaining normal. This case is particularly noteworthy for its extreme clinical severity, characterized by profound neurological impairment and metabolic derangements so refractory that they necessitated emergent renal replacement therapy. We present this case to highlight these rare, life-threatening complications and to discuss the diagnostic challenges of euglycemic ketoacidosis in the setting of GLP-1/GIP receptor agonist therapy.

## Case presentation

History and physical examination

A 45-year-old female with a past medical history of type 2 diabetes mellitus (T2DM) and a previous cerebral infarction was brought to the emergency department (ED) via ambulance after being found unconscious by her family. Her regular medications included metformin, atorvastatin, citalopram, and propranolol. Her medication history was also significant for a structured titration of tirzepatide for weight loss starting in May 2024, which was increased by 2.5 mg every four weeks up to a maximum dose of 15 mg. Due to side effects, the dose had recently been de-escalated to 12.5 mg administered every 10 days. Collateral history provided by the family noted a recent episode of diarrhea and vomiting 10 days prior and a total weight loss of approximately 35 kg over the preceding 10 months.

Upon arrival, the patient was maintaining her own airway. Her respiratory rate was 40 breaths per minute with an oxygen saturation of 100% on room air, and the chest examination was unremarkable. Cardiovascular assessment revealed a blood pressure of 90/40 mmHg and a heart rate of 82 beats per minute, with an unremarkable abdominal examination. The patient's Glasgow Coma Scale (GCS) was 6/15 (E4, V1, M1), with physical examination findings of diffuse bilateral facial and limb muscular rigidity, bilateral nystagmus, and a fixed gaze. She was also found to be profoundly hypothermic with a rectal temperature of 30°C. 

Diagnostic assessment

Given the patient's severe clinical distress, recent history of tirzepatide use, and poor oral intake, there was immediate concern for a profound metabolic derangement. Despite the absence of marked hyperglycemia, the combination of tachypnea and altered level of consciousness raised a strong suspicion for euglycemic ketoacidosis (EKA). Initial venous blood gas (VBG) in the ED confirmed a severe high anion gap metabolic acidosis (HAGMA) with a pH <6.8, a blood glucose level of 4.8 mmol/L, and bedside ketones of 4.7 mmol/L. Further laboratory investigations confirmed acute kidney injury (AKI) stage 3 with a creatinine of 749 µmol/L and an estimated glomerular filtration rate (eGFR) of 5 mL/min/1.73m². Marked lactic acidosis was present (lactate 19.6 mmol/L), alongside an elevated white cell count (WCC) and a significant anion gap, while creatine kinase (CK) levels remained within normal limits. The complete longitudinal trend of these laboratory parameters and their response to intervention are detailed in Table 1.

**Table 1:** Serial Laboratory Parameters and Metabolic Trends From Presentation to Discharge From the Intensive Treatment Unit Abbreviations: pH: potential of hydrogen; pCO2: partial pressure of carbon dioxide; HCO3: bicarbonate; Cl: chloride; Na: sodium; K: potassium; eGFR: estimated glomerular filtration rate; ED: emergency department; RRT: renal replacement therapy; ITU: intensive treatment unit; mmol/L: millimoles per liter; kPa: kilopascals; µmol/L: micromoles per liter. Anion Gap = (Na + K) - (Cl + HCO3).

Blood Results	On arrival to ED	Prior to RRT	24 hours after RRT	Day 10 on ITU	Reference Range
pH	<6.8	6.98	7.42	7.46	7.25 – 7.45
pCO2	3.3 kPa	1.3 kPa	2.1 kPa	4.2 kPa	4.7 – 6.0 kPa
Bicarbonate	Unrecordable (low)	2.5 mmol/L	10.1 mmol/L	22.0 mmol/L	22 – 29 mmol/L
Chloride	100 mmol/L	111 mmol/L	108 mmol/L	116 mmol/L	98 – 107 mmol/L
Anion Gap	-	35.1 mmol/L	20.4 mmol/L	12.0 mmol/L	8 – 16 mmol/L
Glucose	4.8 mmol/L	5.9 mmol/L	6.4 mmol/L	6.6 mmol/L	4.0 – 7.8 mmol/L (random)
Ketones	4.7 mmol/L	4.0 mmol/L	4.4 mmol/L	-	0.0 – 0.6 mmol/L
Lactate	19.6 mmol/L	11.8 mmol/L	7.0 mmol/L	0.9 mmol/L	0.5 – 2.2 mmol/L
Sodium	139 mmol/L	142 mmol/L	135 mmol/L	146 mmol/L	135 – 145 mmol/L
Potassium	5.2 mmol/L	6.6 mmol/L	3.5 mmol/L	4.0 mmol/L	3.5 – 5.0 mmol/L
Urea	16.3 mmol/L	-	6.7 mmol/L	11.3 mmol/L	2.5 – 7.8 mmol/L
Creatinine	749 (µmol/L)	-	249 (µmol/L)	283 (µmol/L)	45 – 90 µmol/L (female)
eGFR	5 (mL/min/1.73m2)	-	19 (mL/min/1.73m2)	17 (mL/min/1.73m2)	>90 mL/min/1.73m²

Treatment and therapeutic intervention

**Figure 1:**
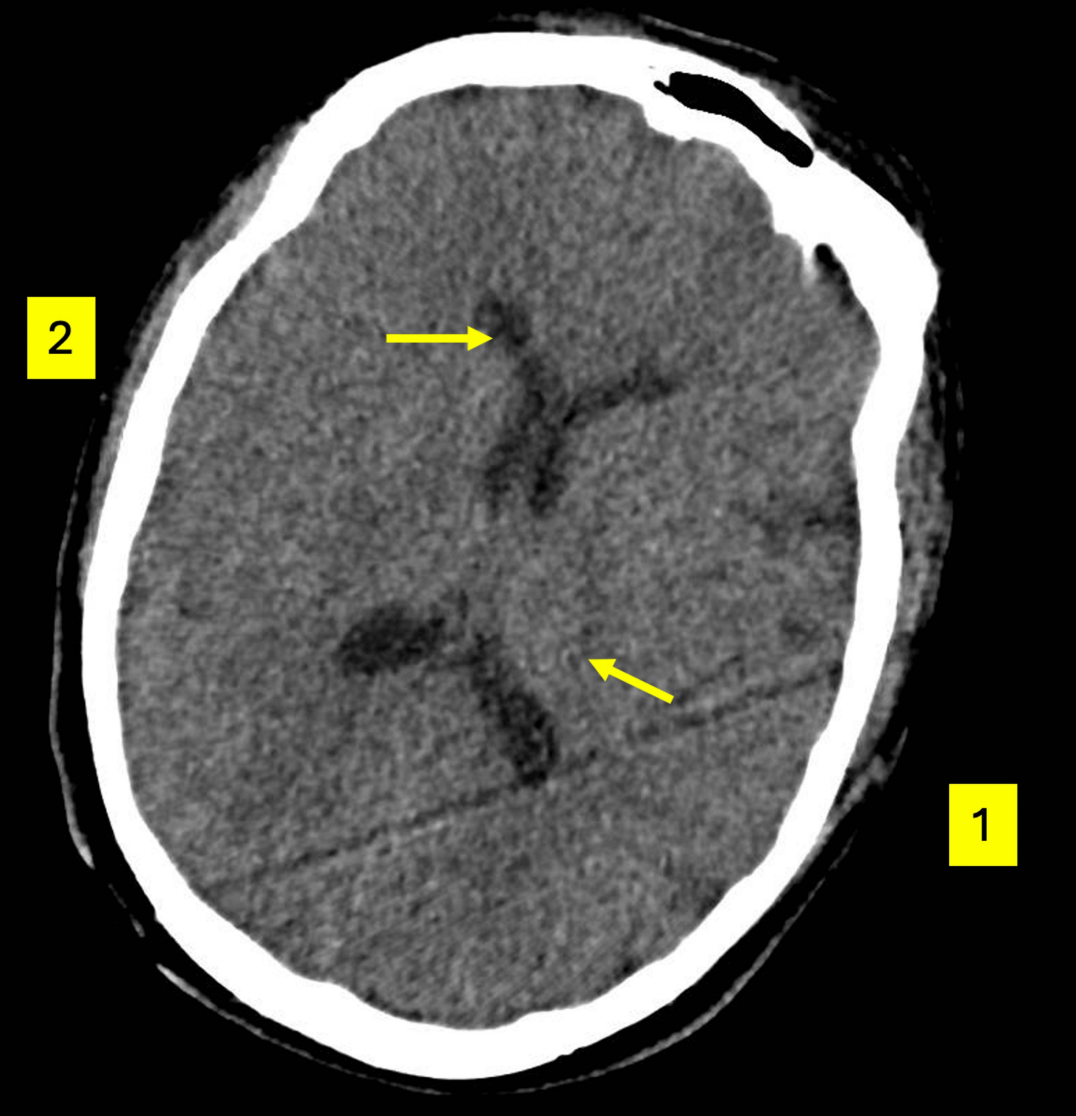
Initial non-contrast axial computed tomography (CT) of the head Axial view at the level of the thalamus is shown. No acute intracranial hemorrhage, midline shift, or large-vessel territory infarction is present. Arrow 1 indicates the right thalamus, and Arrow 2 points to the anterior horn of the right lateral ventricle, both demonstrating normal morphology and attenuation. A diagonal linear streak across the posterior right hemisphere is a motion artifact. Normal pneumatization of the frontal sinus is visible at the anterior aspect of the skull. The absence of structural pathology was critical in excluding primary neurological causes for the patient’s altered sensorium and muscular rigidity, redirecting focus toward a metabolic etiology.

The patient received 6 L of intravenous (IV) 0.9% sodium chloride in the ED without clinical improvement. She was subsequently started on IV thiamine, a fixed-rate intravenous insulin infusion (FRIII), and IV dextrose to manage the euglycemic ketoacidosis (EKA). This combined administration of insulin and dextrose is the cornerstone of EKA management, allowing for the resolution of ketonemia while preventing hypoglycemia [3,5]. Given the neurological findings and hypothermia, empiric IV antibiotics and antivirals were initiated. To further investigate the patient’s reduced GCS and atypical neurological presentation, an urgent non-contrast computed tomography (CT) of the head was performed. This demonstrated no acute intracranial pathology (Figure 1). 

The patient was admitted to the intensive treatment unit (ITU), where she required vasopressor support. Despite aggressive resuscitation with intravenous fluids and insulin infusion, the patient exhibited persistent, refractory HAGMA and worsening hyperkalemia. Given the severity of the biochemical derangement and the presence of acute kidney injury, renal replacement therapy was initiated to facilitate rapid metabolic correction. Acidosis resolved within 24 hours, and ketonemia within 48 hours. On day 2, the patient was intubated to facilitate RRT and manage severe agitation. Despite metabolic normalization, her GCS remained low (3/15) during sedation holds, and limb rigidity persisted for five days. Neurology consultation suggested findings were likely secondary to metabolic derangement, hypoxic-ischemic injury, or potential osmotic demyelination. A lumbar puncture was performed; cerebrospinal fluid (CSF) analysis showed a glucose of 5.1 mmol/L and protein of 0.49 mmol/L. Antimicrobial therapy was discontinued following negative CSF microscopy, culture, and polymerase chain reaction (PCR) testing.

Outcome and follow-up

**Figure 2:**
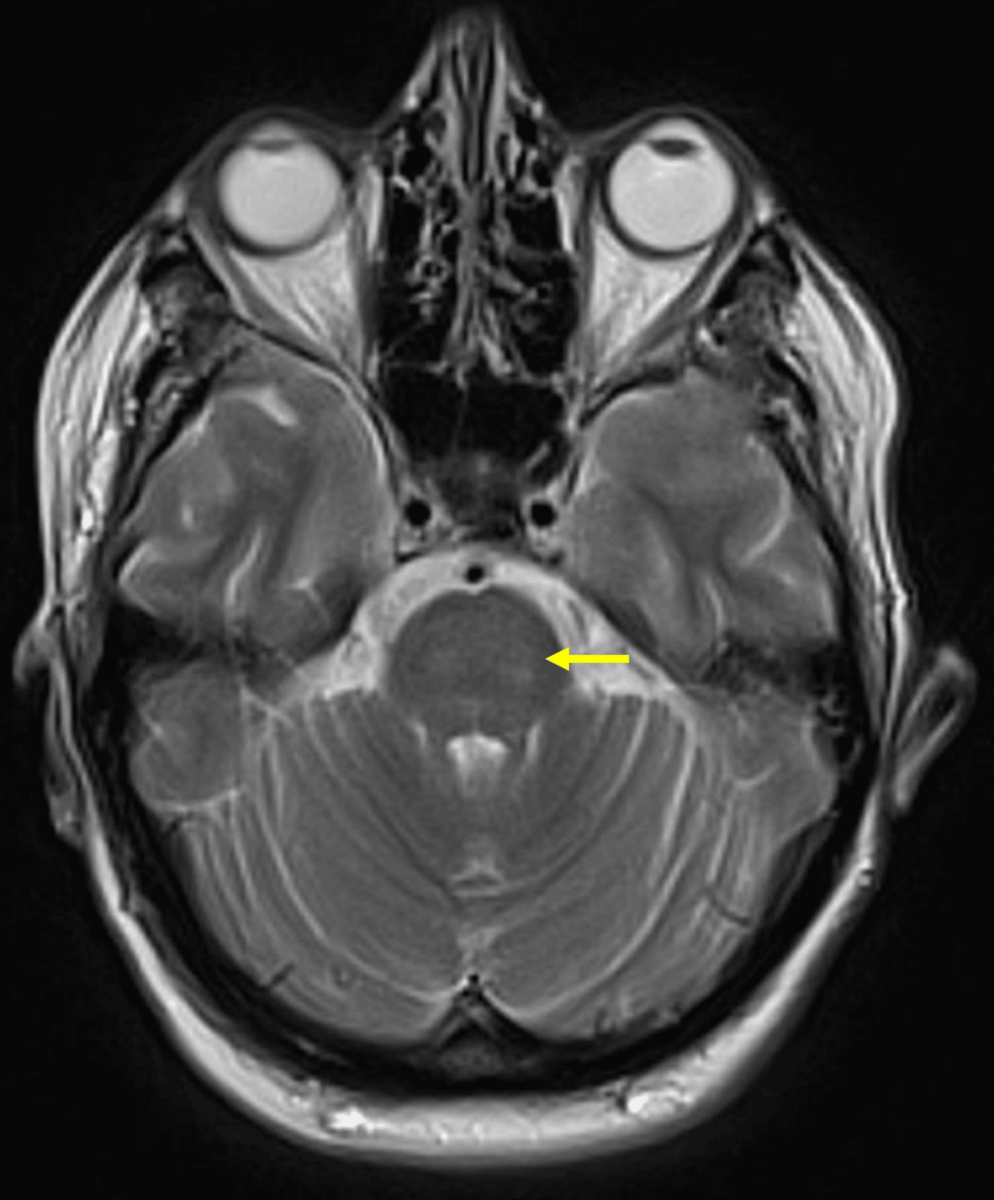
Axial T2-weighted magnetic resonance imaging (MRI) of the brain Axial T2-weighted MRI slice shows a normal, uniform signal within the pons (arrow). There is no evidence of central T2 hyperintensity or the 'Trident sign' characteristic of osmotic demyelination syndrome. This finding effectively ruled out osmotic demyelination as a cause for the patient's prolonged neurological impairment, supporting the diagnosis of severe metabolic encephalopathy secondary to tirzepatide-induced euglycemic ketoacidosis.

The patient’s renal function improved, and she was successfully extubated on day 9 with a GCS of 14/15. She was stepped down to ward-level care on day 12, at which point her muscular rigidity had resolved. An MRI of the brain performed on the ward was reported as normal (Figure 2). Upon further questioning, the patient revealed she had experienced severe nausea and anorexia in the weeks prior to admission, leading to an inability to tolerate oral intake. She was counseled on the risks and subsequently agreed to discontinue tirzepatide.

## Discussion

Dual glucose-dependent insulinotropic polypeptide (GIP) and glucagon-like peptide-1 (GLP-1) receptor agonists work by increasing insulin secretion following an oral glucose load. However, they also delay gastric emptying, reduce appetite, and inhibit glucagon production from pancreatic alpha-cells [6]. Specifically, tirzepatide acts as a dual agonist of these receptors to promote weight loss through these sustained effects on satiety and gastric motility [7]. Additionally, evidence points toward the occurrence of nausea, vomiting, and diarrhea as adverse events in clinical trials studying GLP-1 receptor agonists, particularly in patients also receiving metformin [8]. The synergistic mechanism of action of these medications, combined with these potential gastrointestinal adverse effects, demonstrates that they can put patients at risk of developing euglycemic ketoacidosis (EKA).

In this patient’s case, the use of metformin for type 2 diabetes mellitus (T2DM) may have potentially exacerbated the acidosis due to its link to raised lactate levels during acute illness [9]. The patient’s delayed neurological recovery, despite the resolution of ketoacidosis and uremia, could be linked to tirzepatide. Given that the patient made a complete neurological recovery and had a normal MRI, osmotic demyelination syndrome (ODS) is an unlikely cause. While recent literature has described cases of Wernicke encephalopathy (WE) secondary to tirzepatide-induced nutritional deficiencies [10], the rapid resolution of this patient’s rigidity and fixed gaze suggests a transient metabolic mimic rather than a structural encephalopathy. Therefore, patients who present with persistent high anion gap metabolic acidosis (HAGMA) with normal to low levels of blood glucose and a known history of GIP/GLP-1 receptor agonist use should be suspected of having EKA. Patients diagnosed with T2DM, such as in this case, can be treated with insulin to manage the ketoacidosis and normalize pH levels while maintaining euglycemia with the use of intravenous (IV) dextrose. While some reports of tirzepatide-associated EKA describe milder metabolic derangements that resolve with conservative fluid resuscitation [11], persistent acidosis and anuria despite ongoing medical management may necessitate renal replacement therapy (RRT). This underscores a high-severity end of the clinical spectrum that requires early recognition and aggressive intervention.

## Conclusions

Euglycemic ketoacidosis (EKA) is a well-recognized complication associated with sodium-glucose cotransporter-2 (SGLT-2) inhibitors; however, it is increasingly reported in association with the clinical use of dual GLP-1/GIP receptor agonists such as tirzepatide. This case illustrates a severe presentation of EKA, acute kidney injury, and protracted neurological abnormalities likely precipitated by the gastrointestinal side effects of tirzepatide, which led to significant anorexia and starvation-related ketosis. While a single case report cannot definitively prove causality, it highlights a strong association between dual GLP-1/GIP receptor agonists and potentially life-threatening metabolic derangements in the setting of reduced oral intake. Clinicians should maintain a high index of suspicion for EKA in patients on these medications who present with metabolic acidosis, even in the absence of hyperglycemia. 
